# Novel Targets and Strategies Addressing Residual Cardiovascular Risk in Post-acute Coronary Syndromes Patients

**DOI:** 10.37825/2239-9747.1058

**Published:** 2024-08-28

**Authors:** Francesco P. Cancro, Michele Bellino, Angelo Silverio, Marco Di Maio, Luca Esposito, Rossana Palumbo, Martina L. Manna, Ciro Formisano, Germano Ferruzzi, Carmine Vecchione, Gennaro Galasso

**Affiliations:** Department of Medicine Surgery and Dentistry, University of Salerno, Baronissi, SA, Italy

**Keywords:** Acute coronary syndrome, Secondary prevention, Coronary artery disease, Lipoprotein(a), Inflammation, Inflammasome, Microbiota, Emerging therapies

## Abstract

Despite the advancement in secondary cardiovascular prevention strategies for post-acute coronary syndrome (ACS) patients, the development of new drugs addressing dyslipidemia and the personalization of dual antiplatelet therapies (DAPT), these patients continue to suffer a significant incidence of recurrent ischemic events. Therefore, novel targets that can be tackled to reduce cardiovascular risk are needed to improve the outcome of this very high-risk population.

The role of chronic inflammation and inflammasome in the development and progression of atherosclerosis has been broadly investigated in patients with established coronary artery disease (CAD) and recent randomized trials have highlighted the possibility to manage these targets with specific drugs such as colchicine and monocolonal antibodies with a significant improvement of cardiovascular outcomes in post-ACS patients.

Lipoprotein(a) [Lp(a)] is the most promising non-traditional risk factor and has shown to predict worse outcome in post-ACS patients. Lowering Lp(a) through PCSK9 inhibitors and specific targeted therapies has shown positive results in reducing adverse cardiovascular events in patients with established CAD.

The effect of microbiome and its alteration in gut dysbiosis seems to actively participate in residual cardiovascular risk of CAD patients; however, the risk-modifying effect of targeted-microbiome therapies hasn’t been yet investigated in large population-based studies.

Long-term outcome of post-ACS patients is a complex puzzle of multiple factors.

In this minireview, we summarize the emerging risk factors that may interplay in the residual risk of post-ACS patients and their possible prognostic and therapeutic implications.

## 1. Introduction

Despite continuous advancement in primary and secondary prevention strategies [1–3], improved percutaneous coronary intervention (PCI) techniques [4–6], patients with previous myocardial infarction (MI) continue to experience a significant number of recurrent ischemic events [7–10].

That said, the necessity of targeted intervention in this population raises in order to lower their residual cardiovascular risk, despite optimal guideline-guided therapy [11].

It is clear that mere control of traditional risk factors and lifestyle is not enough to effectively minimize the residual risk of these patients. Therefore, a tailored approach directed at detecting and treating novel and additional risk factors is required to achieve improved short- and long-term prognosis after an Acute Coronary Syndrome (ACS) [12–16].

**Fig. 1:**
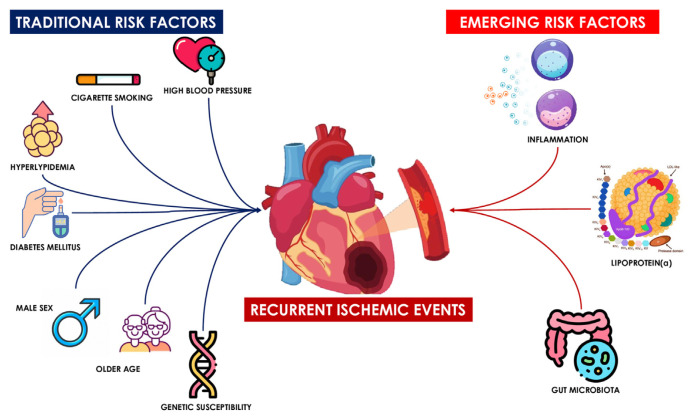
Traditional and novel risk factors for recurrent ischemic events.

The purpose of this mini-review is to summarize the underlying pathophysiological mechanisms of residual cardiovascular risk in post-ACS patients, to outline new risk factors associated with recurrent ischemic events, and to suggest new potential therapeutic targets that may be considered in this very high-risk population (Fig. 1).

## 2. Residual cardiovascular risk after ACS and the limitations of traditional risk factors

In recent years it has become clear that the underlying pathophysiological pathway of ACS is a complex puzzle involving a multitude of elements that leads to the instabilization of atherosclerotic plaque, and the exposure of prothrombotic elements that enhance thrombus formation with the consequential obstruction of the coronary arteries and the development of ACS. This process appears complex since it has been observed in several cases that significantly flow-limiting stable plaques show signs of multiple cycles of rupture and self-healing [17,18]. This assumption has also been corroborated by intravascular imaging studies that have described how morphologically vulnerable plaques could, according to a not yet fully elucidated mechanism, progress to a stable condition, presumably through cycles of rupture and spontaneous healing [19,20]. On the other hand, in some patients, the prothrombotic mechanism outweighs the healing process resulting in the evolution toward an ACS. In these patients, there could be a pathophysiological predisposition that makes them more susceptible to the occurrence of ischemic events. Moreover, this hypothesis has been supported by several clinical studies in which was observed that post-ACS patients had a significantly higher risk of ischemic events than patients with stable CAD, who have not experienced previous ACS [21,22].

Furthermore, the recurrence of ischemic events in post-ACS patients is reported between 4.4% and 6.7%, with an increased risk in the first 12 months post-ACS and a steady increase even in the long term [8,23,24]. These findings reveal how, despite optimal invasive and pharmacological treatments, a significant residual risk persists in this cohort of patients.

The management of residual cardiovascular risk of post-ACS patients needs to consider a complex network of factors. Exogenous factors such as lifestyle habits, diet, daily activities, and biomolecular factors such as thrombotic activity, lipoproteins, and inflammation all intersect and interact with each other. In recent decades, research efforts have focused on reducing thrombotic events with interventions aimed at preventing the instabilization of atherosclerotic plaques through the development of increasingly effective hypolipidemic drugs [25], as well as directly targeting thrombotic activity with the use of antiplatelet drugs and dual antiplatelet therapy (DAPT). The latter, represents the cornerstone of pharmacological therapy in post-ACS, and in recent years DAPT strategies have been adjusted to fit particular cohorts of patients with prohibitively high long-term thrombotic risk [26]. A long-term DAPT strategy with Ticagrelor 60 mg b.i.d. in addition to Aspirin has been tested in the PEGASUS-TIMI 54 (Prevention of Cardiovascular Events in Patients with Prior Heart Attack Using Ticagrelor Compared to Placebo on a Background of Aspirin–Thrombolysis In Myocardial Infarction 54) trial, in which the prolonged DAPT significantly reduced the recurrence of ischemic events in post-MI patients, especially in patients with high atherosclerotic burden, with no difference in terms of fatal and intracranial bleedings [26,27].

The COMPASS (Cardiovascular Outcomes for People Using Anticoagulation Strategies) trial, on the other hand, evaluated an alternative strategy for the prevention of thrombotic events adding to the single antiplatelet therapy with Aspirin a low-dose (2.5 mg b.i.d.) of Rivaroxaban in patients with extensive atherosclerotic disease. This association significantly reduced the incidence of ischemic events, however these patients suffered a higher risk of major bleedings [28].

Given these data, it is essential to adopt a tailored approach for each patient, considering both their thrombotic and hemorrhagic risk, to select the most suitable therapy [29–31].

In this setting LDL-C is a well-proven causative contributor to CAD [32]. Although the introduction of drugs such as statins and ezetimibe have markedly reduced the incidence of ischemic events in patients with CAD [33,34], significant residual risk persists in this patient population even with maximal hypolipidemic therapies [35]. This problem was mitigated with the introduction of proprotein convertase subtilisin/kexin type 9 (PCSK9) inhibitors, which succeeded in further reducing cardiovascular events in patients who failed to reach LDL-C targets despite conventional therapies [36–38]. To seek additional factors associated with residual risk in these patients, several subanalyses of randomized trials have focused on finding further predictors of events [39,40]. In particular, a subanalysis of the FOURIER (Further Cardiovascular Outcomes Research With PCSK9 Inhibition in Subjects With Elevated Risk) trial showed that despite optimally controlled LDL-C levels with PCSK9 inhibitors, high-sensitivity C-reactive protein (hsCRP) was a significant predictor of long-term events [39]. In addition, a prespecified analysis of the ODYSSEY Outcomes study showed that Lipoprotein(a) [Lp(a)] was a strong predictor of long-term ischemic events, independently of LDL-C values [41].

This evidence suggests that there is still a way to go in optimizing the residual cardiovascular risk of patients with CAD and that the aim of future research is to focus on and treat emerging risk factors.

## 3. Inflammation and atherosclerosis

Inflammation plays a central role in the development and progression of atherosclerosis [42].

Traditional risk factors such as hypertension, cigarette smoking, hyperlipidemia and diabetes can activate endothelial damage and dysfunction promoting a vascular low-grade inflammatory response, leading to the progression of atherosclerosis [43]. On this basis, inflammation represents a common mechanism bridging traditional and emerging CV risk factors to the development of atherosclerosis [44]. Specifically, it has been observed that LDL can activate T cells [45]. Furthermore, hypertension also appears to be linked to inflammatory patterns, as angiotensin II promotes the induction of nuclear transcriptors related to inflammatory response [46]. Moreover, several studies have previously reported that excess visceral adipose tissue associated with insulin resistance can act as a trigger for the release of proinflammatory cytokines [47]. Finally, cigarette smoking is known to correlate with increased inflammatory stimulus. In fact, besides correlating with an increase in leukocytes, an increase in inflammatory markers correlated with smoking has also been observed [48].

Recently, improved outcomes of patients with CAD receiving anti-inflammatory therapies have ignited the debate on the importance of this pathophysiological process in the residual risk of these patients [49–52]. Atherosclerosis is at the core of a chronic vicious circle in which inflammatory cells penetrate the damaged endothelium and there release several proinflammatory cytokines [53–56]. In this environment, the persistence of necrotic endothelial cells, which compose the necrotic core of the atherosclerotic plaque, causes a further activation and promotion of the inflammatory process, leading to a chronic damaging self-fed process [57,58].

Furthermore, a subanalysis of CANTOS (The Canakinumab Anti-Inflammatory Thrombosis Outcomes Study) trial showed a significant lower incidence of ischemic events in post-MI patients with lower hsCRP levels treated with Canakinumab [59]. Moreover, a continuous association between CRP levels and recurrence of CAD has been described in a large metanalysis involving 160,309 subjects from 54 long-term prospective [60].

Recently, inflammasome has been extensively investigated as one of the pivotal regulatory factors in the inflammatory process [61,62], and, considering this evidence, the inflammasome nucleotide-binding oligomerization domain, leucine-rich repeat-containing receptor (NLR) family pyrin domain-containing 3 (NLRP3) has been broadly analyzed and characterized as being considered one of the key players in atherosclerosis [62].

Cholesterol crystals from atherosclerotic plaque, Ox-PLs, and endothelial wall shear stress appear to be the main triggers of NLRP3 activation [63–65]. When activated, NLRP3 promotes the activation of inerleukin-1β (IL-1β), which possesses numerous proinflammatory effects, stimulating the secretion of additional cytokines and interleukins, through which in addition to sustaining the inflammatory process, also promotes the release of acute-phase factors such as fibrinogen and plasminogen activator inhibitor-1 (PAI-1), promoting the formation of a prothrombotic environment [66–69].

In several studies, elevated IL-1 has been associated with worse ischemia-reperfusion injury as well as increased negative cardiac remodelling while, its inhibition, has been related to decreased negative remodelling and lower release of acute phase factors [70–72].

Based on this evidence, the inflammatory cascade emerges as a crucial factor in the progression of atherosclerosis in patients with CAD, as well as a potential predictor contributing to the residual risk of such patients.

### 3.1. Anti-inflammatory therapies for CAD patients

Targeting NLRP3 inflammasome and other mediators of inflammation represent an interesting area of research and seems to be a future paradigm in treatment of post-MI patients.

Colchicine is one of the most promising anti-inflammatory drugs used in this type of patient. European Society of Cardiology (ESC) 2021 guidelines on cardiovascular disease prevention suggest that low-dose colchicine (0.5 mg daily) may be considered in secondary prevention, especially if other risk factors are insufficiently controlled or if recurrent cardiovascular events occur under optimal therapy (IIb) [73]. This indication has been based on the results of two randomized trials, COLCOT and LoDoCo2, in which low-dose colchicine was shown to be effectively reducing cardiovascular events in patients with CAD [51,52]. Furthermore, a recent meta-analysis that pooled 11 randomized studies on the use of colchicine in patients with CAD reported a significant reduction in both long-term cardiovascular and cerebrovascular events [74].

Methotrexate (MTX), a widely used antirheumatic drug, has also been tested in patients with CAD. Specifically, a meta-analysis performed including 10 observational studies had previously described a significant reduction in cardiovascular events in patients with rheumatoid arthritis treated with MTX [75]. This finding, though, has not been confirmed in the Cardiovascular Inflammation Reduction trial (CIRT), in which no significant reduction in cardiovascular events was reported in patients with atherosclerotic disease receiving low-dose MTX [76]. The negative result of this study could be related to the drug formulation, therefore a new study investigating the effect of MTX formulated in LDL nanoparticles is currently ongoing and results are expected in the next few years (NCT04616872).

Among immunomodulatory monoclonal antibodies, Canakinumab, administered at a dose of 150 mg every 3 months subcutaneously, has been found to significantly reduce cardiovascular events compared with placebo in the CANTOS study [50]. In addition, in a subanalysis of the same study, a reducing effect on proinflammatory factors, specifically IL-6 and hsCRP, was also documented [59]. Those effects appear to be directly related to its action as a hindrance between IL-1β and its receptor. However, it should be considered that the immunomodulatory effect of this molecule is associated with significant infectious-immunologic adverse effects since the action of this molecule occurs systemically [50].

Two recent randomized trials (ASSAIL-MI and RESCUE) tested the effect of further monoclonal antibodies, Tocilizumab and Ziltivekimab, which antagonize IL-6 and IL-6 ligand, respectively [77,78]. Specifically, Tocilizumab at a dose of 280 mg demonstrated an increase in myocardium salvage after revascularization in patients with STEMI [77], while Ziltivekimab was effective in reducing plasma values of inflammatory and thrombotic biomarkers in the context of advanced renal disease [78].

Finally, two new molecules that can directly target NLRP3 inflammasome, namely arglabin and MCC950, have been shown in several experimental animal models to effectively reduce inflammatory triggers, improve lipid profile, reduce aterosclerotic plaque size, and reduce the area of myocardial infarction [49,79,80]. However, clinical studies in humans are awaited to evaluate the actual benefit of these molecules.

Although anti-inflammatory therapy may represent a future ace in the hole in secondary prevention for patients with CAD, the potential adverse reactions and the cost of such drugs make this option currently extremely limited. Future research should focus on developing effective and safe molecules with affordable costs for patients and national health services.

Table 1 summarizes the emerging antinflammatory therapies.

## 4. Lipoprotein(a): the “new” actor in atheroscleorosis

Lipoprotein(a) [Lp(a)] is a plasma lipoprotein composed of an LDL-rich particle and an apolipoprotein B100 molecule bound via a single disulphide link to apolipoprotein(a) [apo(a)], a plasminogen-like glycoprotein [81]. ^43^ Its plasma levels are mostly genetically determined and varies from <0.1 mg/dL to >300 mg/dL (<0.2–750 nmol/L), generally (>90% of the cases) with no significant influence from dietary and environmental factors [81].

Lp(a) has been described as a supplemental actor in the pathogenesis of atherosclerosis, participating in foam cell formation, smooth muscle cell proliferation, inflammation, and plaque instability [82]. These effects are not only related through similar LDL mechanisms, but also to specific aop(a)-related pathways. Apo(a) exhibits a high affinity for damaged vascular endothelium due to lysine binding sites that interact directly with the exposed portions of endothelium, allowing its subintimal entry and deposition [83]. In addition, apo(a) can actively interfere in the fibrinolysis process and promote a prothrombotic status due to its plasminogen-like structure [84].

In addition, Lp(a) promotes atherogenosis and inflammation by behaving as a carrier for Ox-PLs within the endothelium [85]. In fact, in a study involving patients with elevated Lp(a) levels, excessive accumulation of 18-fluorodeoxyglucose within the vascular walls, itself an indication of inflammation, was documented, with additional increased reactivity of inflammatory cells [86].

### 4.1. Lipoprotein(a) in cardiovascular prevention

Over the past years, several primary prevention studies conducted in healthy patients with no history of atherosclerosis have shown that plasma Lp(a) levels exhibit a linear and independent association in the development of atherosclerotic cardiovascular disease (ASCVD) [87–91]. The Copenhagen City Heart Study showed an increased risk of cardiovascular events in healthy subjects, linearly associated with plasma Lp(a) levels at a 10-year follow-up, especially for subjects with extremely high Lp(a) plasma levels [87]. In an individual patient data meta-analysis including 7 randomized clinical studies, a linear association has been observed between the risk of cardiovascular events and plasma Lp(a) levels, independently of C-LDL values and with hypolipidemic drugs treatments [92]. Moreover, both Lp(a) and small isoforms of apo(a) have emerged as independent causal factors of CAD in a recent Mendelian randomization study involving nearly 17 thousand patients [93].

This evidence affirmed Lp(a) as an independent risk factor for atherosclerotic cardiovascular disease, driving exponential interest in the development of therapies that could impact its plasma levels and activity. Noteworthy, measuring Lp(a) at least once in adults, preferably in the first lipid profile has been recommended by the European Society of Cardiology (ESC) and European Atherosclerosis Society (EAS), with a desirable plasma level <30 mg/dL [94].

Several studies have recently evaluated the role of Lp(a) in the secondary prevention of patients with established CAD [7,40,95–102].

The Copenhagen General Population Study described that patients with established ASCVD and with high Lp(a) levels (≥ 50 mg/dL) suffer a higher risk of major cardiovascular events (MACE), regardless of C-LDL levels [103]. Moreover, a subanalysis of the ODISSEY Outcomes trial, which evaluated the effect of alirocumab vs. placebo in post-MI patients, found an independent linear association between Lp(a) plasma levels and risk of MACE and, furthermore, the reduction on Lp(a) plasma levels provided by alirocumab was significantly associated with a reduction in long-term MACEs [40]. Furthermore, a real-world observational study conducted on 12064 post-PCI described a significant and independent association between high Lp(a) levels and the long-term risk of repeated revascularization [102]. Finally, Lp(a) levels seems to better predict the risk of recurrence of ischemic events in patients without history of diabetes [99].

These data yield robust evidence for the consideration of Lp(a) as an integrative prognostic factor in patients with estabilished CAD and highlight the emergent need toward the development of therapies that will be effective in reducing its plasma levels.

### 4.2. Current and emerging therapies for high lipoprotein(a)

To date, there are no approved pharmacological agents targeting specifically Lp(a) nor preliminary data from randomized clinical trial on Lp(a) reduction and, the only effective treatment is lipoprotein apheresis, which is an invasive procedure, suitable only for secondary prevention in patients with extremely high Lp(a) levels and recurrent cardiovascular events despite optimal medical therapy [104].

Statins, a cornerstone of dyslipidemia treatment, still they have not shown any benefit in reducing Lp(a) plasma levels [105]. Conversely, niacin has shown in the AIM-HIGH (Atherothrombosis Intervention in Metabolic Syndrome with Low HDL/High Triglyceride and Impact on Global Health Outcomes) study a discrete reduction in Lp(a) values of about 20% without, however, significantly decreasing the cardiovascular risk of the enrolled patients [97].

Different phase 3 trials have found a positive effect of mipomersen (an antisense oligonucleotide targeting apolipoprotein B synthesis) in reducing Lp(a) levels by 26%, however its effect on the prognosis in patients with CAD has not yet been tested [106].

Data from the randomized trials FOURIER and ODISSEY Outcomes, which tested the effect of PCSK9 inhibitors in patients with established CAD, respectively of evolocumab and alirocumab, showed not only a significant reduction in Lp(a) levels in these patients, but also a clinical benefit in terms of reduction of long-term cardiovascular events, independently from other serum lipoproteins [41,101].

Furthermore, in the ORION-10 and ORION-11 trials, Inclisiran, a small-interfering (siRNA) PCSK) inhibitor, showed a positive effect in decreasing Lp(a) plasma levels; however its clinical impact has not yet been tested in secondary prevention patients [107].

Moreover, ESC/EAS guidelines recommends the use of PCSK9 inhibitors in patients with familial hypercholesterolemia and high Lp(a) (IIa) [108].

Finally, novel apo(a)-specific target therapies are being investigated through Pelacarsen and Olpasiran drugs (phases II and III), which have exhibited favorable results in terms of reducing Lp(a) plasma values, while prognostic outcome data are expected in the forthcoming years [109–111].

Table 2 summarizes the emerging lowering Lp(a) therapies.

## 5. Gut microbiota

Gut microbiota is composed of several microbes colonising the whole digestive tract and its composition and functioning could be affected by genetic, dietary, and environmental factors [112,113]. Innate immunity is constantly engaged in compensating the chronic inflammatory trigger generated by microbiota agents themselves [114]. The microbiota’s composition could be deeply impaired by a condition known as gut dysbiosis, which could take part in the pathogenesis of several intestinal and non-intestinal diseases such as diabetes, obesity, and cerebrovascular diseases [115]. Recently, it has been investigated that alterations in the microbiota may promote the crossing within the systemic circulation of particular bacterial products such as lipopolysaccharide (LPS) and trimethylamine-N-oxide (TMAO) which may trigger atherothrombotic and ischemic cardiovascular events [73,116]. However, controversial results have been brought from small clinical studies investigating the association between TMAO and atherosclerotic cardiovascular events and its effect in atherosclerosis has been observed only in animal models [117–122].

Considerable interest, however, is growing on the possibility that LPS may play a role in the development of ASCVD through a low-grade endotoxemia induced by itself [123]. Due to its interaction with toll-like receptors, which are located on several cellular walls, it has been associated with the development of thrombotic phenomena [124]. Moreover, studies conducted in healthy subjects, highlighted the association between the soluble levels of LPS and the risk of ASCVD [125–127]. An enhanced gut permeability has been observed in patients suffering a MI and circulating levels of LPS were associated with thrombus burden in patients with STEMI in a small case-control study [128]. Recently, an observational study of post-STEMI patients found that LPS circulating levels occurring during MI were significantly associated with the long-term risk of adverse cardiovascular events [129]. However, current evidence on the role of dysbiosis and bacterial proteins in predicting future adverse events in patients with CAD is strongly limited, and their role in prognostic stratification of these patients remains unclear.

Fiber-rich diets such as the Mediterranean diet, the use of prebiotics and probiotics, and fecal microbiome transplants have been demonstrated to be beneficial in positively regulating intestinal permeability and in lowering the circulating levels of bacterial products. However, these effects have not yet been analyzed in randomized clinical trials or studies with strong statistical power [116,123].

## 6. Conclusions and future perspectives

Despite the optimization of secondary prevention strategies, and the approval of new drugs, post-ACS patients still have a non-negligible risk of long-term adverse events. The residual risk of these patients is affected by several interacting factors forming a complex pathophysiological patchwork. A wide-ranging and tailored approach is required to minimize the occurrence of long-term adverse events.

The chronic inflammatory stimulus represents one of the key players in the atherosclerotic process, and recent studies on the effect of anti-inflammatory drugs on cardiovascular risk have yielded promising results. However, the safety profile of these drugs and their cost-effectiveness is still to be clarified.

Lp(a) is one of the most promising emerging risk factors, and several studies have established its long-term prognostic value. Several studies are ongoing to develop specific drugs to influence its plasma values and pathogenic activity.

The study of gut microbiome and the effects of dysbiosis is a field that is gaining considerable interest in different clinical settings. Its correlation with atherothrombotic events and the possibility of targeting it could represent a breakthrough for the future therapies of these patients.

Finally, the future cardiology should increasingly focus on seeking out peculiar factors that may contribute to the residual risk of patients with CAD with the goal of specifically targeting them.

## Figures and Tables

**Table 1:** Anti-inflammatory and immune therapies in patients with established CAD.

Drug	Principal mechanism of action	Study	Findings
Colchicine	Inhibition of microtubules polymerisation	COLCOT (NCT02551094) LoDoCo2 (ACTRN12614000093684)	Colchicine 0.5 mg significantly reduced the risk of ischemic CV events
Canakinumab	Anti-IL-1β monoclonal antibody	CANTOS (NCT01327846)	Canakinumab 150 mg every 3 months significantly reduced the rate of recurrent CV events
Methotrexate	Folate pathway antagonist	CIRT (NCT01594333)	MTX 15–20 mg weekly did not reduce levels of IL-1β, IL-6, or CRP and did not reduce CV events; a phase III RCT evaluating MTX delivered in LDL-nanoparticle is ongoing (NCT04616872)
Anakinra	Inhibition of the interaction between IL-1 and IL-1R	VCU-ART3 (NCT01950299)	Anakinra significantly reduces the systemic inflammatory response after STEMI
Tocilizumab	Inhibits IL-6R	ASSAIL-MI (NCT03004703)	Tocilizumab increased myocardial salvage; no difference in terms of CV outcomes
Ziltivekimab	Inhibits IL-6 ligand	RESCUE (NCT03926117)	Ziltivekimab reduced biomarkers of inflammation and thrombosis; phase III RCT evaluating the effect on CV outcomes is ongoing (NCT05021835)

CAD, coronary artery disease; CKD, chronic kidney disease; CRP, C-reactive protein; CV, cardiovascular events; hs-CRP, high-sensitivity C-reactive protein; IL-1, interleukin-1; IL-1R, interleukin-1 receptor; IL-1β, interleukin-1β; IL-6, interleukin-6; IL-6R, interleukin-6 receptor; LDL, low density lipoprotein; MI, myocardial infarction; MTX, methotrexate; MVD, multivessel disease; STEMI, ST-elevation myocardial infarction; Pts, patients; RCT, randomized controlled trial.

**Table 2:** Emerging therapies for lowering Lp(a) serum level.

Drug	Principal mechanism of action	Mechanism of Lp(a) lowering	Study	Findings
Evolocumab	Monoclonal antibody inhibiting LDL-R degradation by targeting PCSK9	Inhibition of apo(a) secretion	FOURIER (NCT01764633)	Evolocumab significantly reduced Lp(a) by a median of 26.9%; Evolocumab reduced the risk of death, MI, or PCI by 23% in patients with a baseline Lp(a) > median value.
Alirocumab	Monoclonal antibody inhibiting LDL-R degradation by targeting PCSK9	Inhibition of apo(a) secretion	ODISSEY Outcomes (NCT01663402)	Alirocumab significantly reduced Lp(a) by 23%; Alirocumab independently reduced the risk of CV adverse outcomes.
Inclisiran	siRNA inhibiting LDL-R degradation targeting PCSK9	Inhibition of apo(a) secretion	ORION-10 ORION-11 (NCT03399370; NCT03400800)	Inclisiran reduces Lp(a) plasma levels by 19–22%; The effects on CV outcomes is unknown.
Mipomersen	ASO inhibiting apo(B) synthesis	–	Four phase III trials (NCT00607373; NCT00706849; NCT00770146; NCT00794664)	Mipomersen reduced Lp(a) plasma levels from 20% to 40%; The effects on CV outcomes is unknown.
Olpasiran	siRNA targeting apo(a) mRNA and leads to degradation	–	OCEAN[a]-DOSE (NCT04270760)	Olpasiran reduced Lp(a) plasma levels from 67% to 97%; The effect on CV outcomes is unclear.
Pelacarsen	ASO targeting apo(a) mRNA and leads to degradation	–	AKCEA-APO(a)-LRx (NCT03070782)	Pelacarsen reduced Lp(a) plasma levels by 80%. A phase III RCT evaluating the effect on CV outcomes is ongoing (NCT04023552)

ACS, acute coronary syndromes; Apo(a), apolipoprotein(a); Apo(B), apolipoprotein(B); ASCVD, atherosclerotic cardiovascular disease; ASO, antisense oligonucleotide; CAD, coronary artery disease; CV, cardiovascular; CVD, cardiovascular disease; HC, hypercholesterolemia; HeFH, heterozygous familial hypercholesterolemia; HoFH, homozygous familial hypercholesterolemia; LDL-R, low density lipoprotein receptor; Lp(a), lipoprotein(a); MI, myocardial infarction; PCI, percutaneous coronary intervention; PCSK9, Proprotein convertase subtilisin/kexin type 9; Pts, patients; RCT, randomized controlled trial; siRNA, small-interfering RNA.
